# Proteomic Analysis of Midgut of Silkworm Reared on Artificial Diet and Mulberry Leaves and Functional Study of Three *UGT* Genes

**DOI:** 10.3390/ijms26031309

**Published:** 2025-02-04

**Authors:** Shengxiang Zhang, Xinran Zhang, Jiawen Liang, Shuxian Huang, Bokai Huang, Chunjiu Ren, Huiju Gao, Qingxin Liu

**Affiliations:** 1 College of Forestry, Shandong Agricultural University, Taian 271018, China; zhangxinran08@163.com (X.Z.); 15621317420@163.com (J.L.); 19195673543@163.com (S.H.); hbk202414514191981@163.com (B.H.); ghj@sdau.edu.cn (H.G.); 2 State Key Laboratory of Crop Biology, College of Life Sciences, Shandong Agricultural University, Taian 271018, China; liuqingxin@sdau.edu.cn

**Keywords:** silkworm, artificial feed, proteomics, UDP-glucose glucosyltransferase, molecular docking

## Abstract

There remains a significant gap in production performance and disease resistance between silkworms reared on artificial diets and those reared on mulberry leaves. This study aims to identify key differential proteins through proteomic analysis of the midgut of silkworms fed artificial diets compared to those fed mulberry leaves. Utilizing molecular docking technology, three anti-nutritional factors that consistently bind to the UGT40B4, UGT340C2, and UGT40A1 proteins were selected, and the differential expression of these UGT genes in response to various anti-nutritional factors was examined. The findings indicate that variations in feed significantly influence the expression of digestive, metabolic, and immune-related proteins within the silkworm midgut. Notably, the expression levels of the UGT40B4, UGT340C2, and UGT40A1 genes vary across different silkworm organs and developmental stages, reflecting their respective physiological roles. Furthermore, the effects of soybean isoflavone, tannic acid, and arabinoxylan on silkworm growth and cocoon quality were found to differ when these substances were incorporated into semi-synthetic feed. This research is anticipated to provide valuable insights for future studies on the role of UGT genes in the silkworm midgut and the formulation of artificial diets for silkworms.

## 1. Introduction

The silkworm is not only an economically significant insect with favorable genetic traits but is also considered an ideal lepidopteran model for scientific research [1,2,3]. As an oligophagous insect, the silkworm primarily feeds on fresh mulberry leaves, from which it derives all necessary nutrients and water [4,5,6]. This relationship is a result of long-term co-evolution and natural selection between silkworms and mulberry trees. Although artificial diets for silkworms replicate the composition of mulberry leaves, the consumption of artificial feed varies significantly among different silkworm varieties compared to that of mulberry leaves [7]. Consequently, issues related to weak silkworm physique, silk protein synthesis, and low silk yield remain unresolved [8].

At the end of the 20th century, proteomics research technology began to be applied to silkworm research. With the advancement of the silkworm genome project, silkworm proteomics research has also become the focus of silkworm biology research [9,10]. Studies have revealed the phosphorylation difference of the N-terminal sequence of the silk protein heavy chain molecule through proteomics analysis and found that the Filippi’s gland affects the silk viscosity during the spinning process of the silkworm by regulating the post-translational modification [11]. The immune mechanism of the silkworm fat body against Bacillus cereus ZJ-4 was studied by proteomics. The results showed that the differentially expressed proteins were mainly involved in stress response, biological regulation, and innate immunity. Bacillus cereus ZJ-4 can destroy the innate immune pathway of silkworms and affect the normal immune function of fat cells [12].

UDP-glycosyltransferases (UGTs) are a superfamily of proteases related to glycosylation, which are ubiquitous in animals, plants, bacteria, and viruses [13,14,15,16]. In insects, UGTs play an important role in many processes, including detoxification of substrates such as plant allelochemicals, cuticle formation, pigmentation, and olfactory function [17]. Studies have shown that UDP-glycosyltransferase (UGT) catalyzes a series of different lipophilic small compounds to combine with sugars to produce glycosides, which plays an important role in the detoxification of xenobiotics and the regulation of insect endostatin. Many *UGTs* are expressed in fat bodies, midgut, and Malpighian tubules, indicating that they play a role in detoxification. Some *UGTs* are expressed in antennae, indicating that they play a role in pheromone recognition [18]. A total of 42 *UGT* genes were found in the silkworm genome. Through gene chip technology and RT-qPCR analysis, the results showed that different *UGT* gene expression patterns were different. The *BmUGT013829* gene can cause glycosylation of flavonoids, and also participate in olfactory and detoxification reactions [17]. Another study showed that *BmUGT10295* and *BmUGT8453* genes were significantly expressed in the midgut and Malpighian tubules of the silkworm infected by *N.bombycis*. After overexpression of the two genes, the number of microparticles in the samples was significantly reduced, while after RNAi interference gene expression, the number of microparticles in the samples was significantly increased, indicating that the two genes were induced to express and have resistance to microparticle disease [19]. The glycosylation of UDP-glucosyltransferase (UGT) is of great significance in controlling and eliminating endogenous and exogenous toxins. *BmUGT10286* (*UGT86*) can directly affect the formation of green pigments in silkworm cocoons. The expression of *UGT86* is not only expressed in digestive ducts and silk gland tissues but also in Malpighian tubules, fat body tissues, and gonads [20]. UDP-glucosyltransferase and ABC transporters are involved in substance metabolism and detoxification processes [21].

In this study, iTRAQ (isobaric Tags for Relative and Absolute Quantitation) technology was utilized to investigate the proteomics of the silkworm midgut, comparing specimens reared on an artificial diet versus those fed mulberry leaves, leading to the identification of significantly differential proteins. Through molecular docking technology, three anti-nutritional factors were identified that stably bind to the *UGT40B4*, *UGT340C2*, and *UGT40A1* proteins. We further explored the impact of these distinct anti-nutritional factors on the expression and activity of the three *UGT* genes. This research is anticipated to lay a theoretical foundation for future investigations into the functional roles of *UGT* genes within the silkworm midgut and contribute to the development of optimized artificial diets for silkworm rearing.

## 2. Results

### 2.1. Proteome Differential Expression Analysis

In the midgut samples of artificial diet-rearing silkworms and mulberry leaf-rearing silkworms, we screened them with Fold change > 1.2 and Q value < 0.05. The results showed that there were 564 up-regulated proteins and 400 down-regulated proteins in the midgut of artificial diet-rearing silkworms compared with mulberry leaf-rearing silkworms. There were 964 significantly different proteins.

### 2.2. Statistical Analysis of Differential Protein KEGG Classification

**Figure 1:**
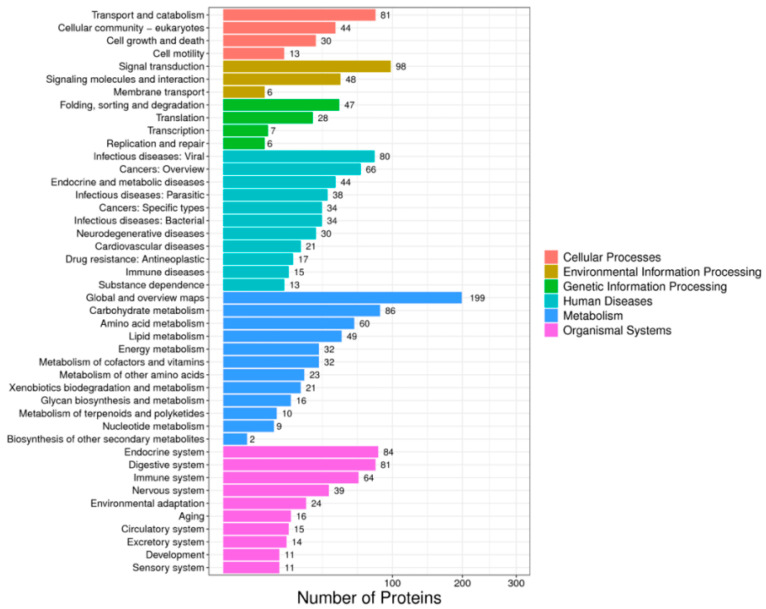
KEGG Pathway classification of differentially expressed proteins.

KEGG (Kyoto Encyclopedia of Genes and Genomes) is a tool mainly used to study the interaction between metabolic pathways, genes and proteins. By analyzing the KEGG pathways involved in differential proteins, we found that they include six categories: cellular processes, environmental information processing, genetic information processing, human diseases, metabolism and organic systems (Figure 1). In these six categories, they are involved in transport and catabolism, signal transduction, transcription, viral diseases, carbohydrate metabolism and endocrine system pathways.

### 2.3. KEGG Enrichment Analysis of Differential Proteins

**Figure 2:**
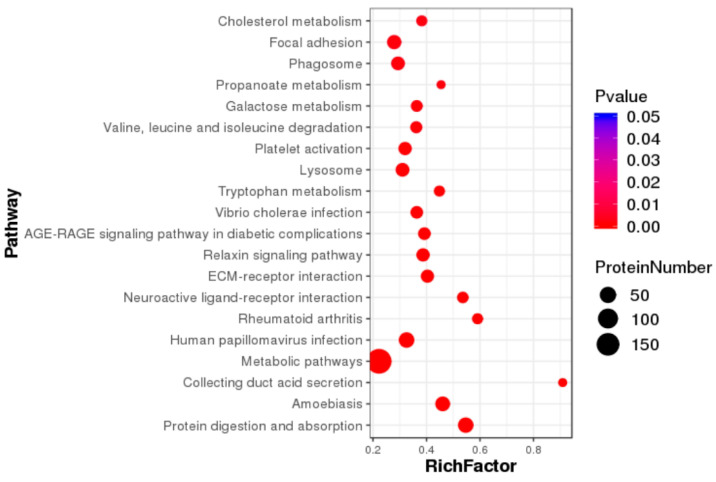
KEGG pathway enrichment bubble map of differentially expressed proteins.

Through the analysis of differential proteins, it was found that they were mainly enriched in metabolic pathways, protein digestion and absorption, lysosome, propionic acid metabolism, galactose metabolism, valine, leucine and isoleucine degradation, phagosome, tryptophan metabolism and other pathways. Among them, 197 differentially expressed proteins were enriched in metabolic pathways, 41 differentially expressed proteins were enriched in protein digestion and absorption pathways, 34 differentially expressed proteins were enriched in protein processing pathways in endoplasmic reticulum, 27 differentially expressed proteins were enriched in lysosomal pathways, and 24 differentially expressed proteins were enriched in PI3K-Akt pathway. A total of 24 differentially expressed proteins were enriched in the oxidative phosphorylation pathway (Figure 2).

### 2.4. RT-qPCR Validation Analysis

**Figure 3:**
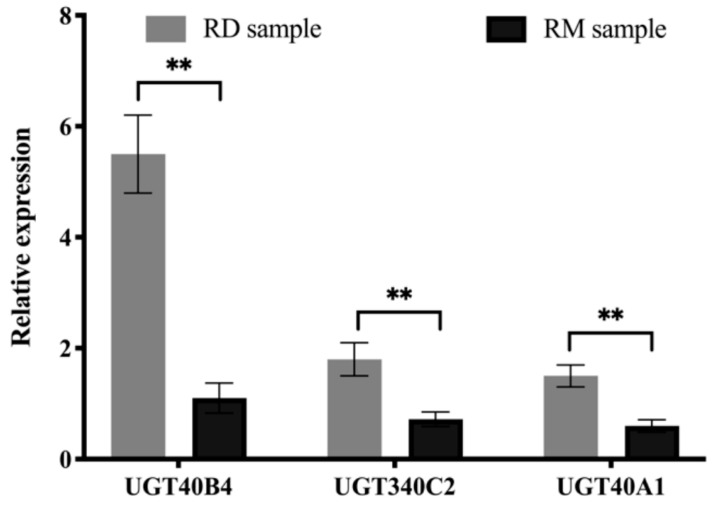
Transcript level validation of *UGT40B4* (AEW43167.1), *UGT340C2* (AEW43159.1) and *UGT40A1* (AEW43163.1). Mean and standard deviation were used for evaluation, the sample size was three (** *p* < 0.01; two-tailed *t*-test).

Through proteomics analysis and fluorescence quantitative analysis, *UGT40B4* (AEW43167.1), *UGT340C2* (AEW43159.1) and *UGT40A1* (AEW43163.1) proteins in the uridine diphosphate glucose transferase family were screened out. They are involved in carbohydrate metabolism, lipid metabolism, cofactor and vitamin metabolism, biodegradation and metabolism of xenobiotics and other pathways. The expression levels of these three proteins in the midgut of artificial diet-rearing silkworms at the transcriptional level and protein level were significantly higher than those of mulberry leaf-rearing silkworms (Figure 3).

### 2.5. UGT Gene Expression Profile Analysis

**Figure 4:**
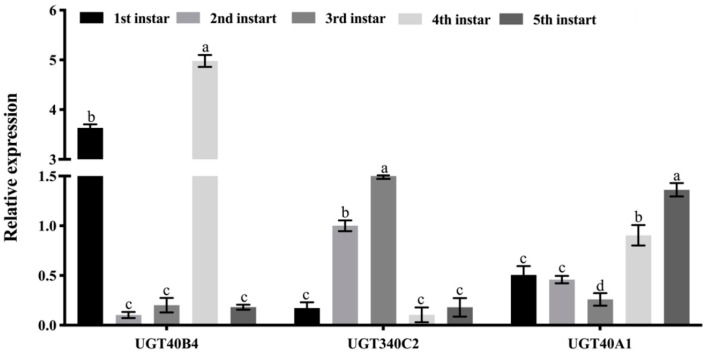
*UGT* gene age expression profile. Data represent three biological repetitive mean +/− standard deviation, according to Duncan’s multiple range test, to list the different letter’s mean significant difference (*p* < 0.05).

Through the analysis of the instar expression profile and tissue expression profile of the three *UGT* genes, it was found that in the instar expression profile, the relative expression of *UGT40B4* gene was higher in the 1st and 4th instars of the silkworm (Figure 4). The relative expression level of *UGT340C2* gene gradually increased from the 1st to 3rd instars of the silkworm, and the relative expression levels of the 2nd and 3rd instars were significantly higher, and the relative expression levels of the 4th to 5th instars were lower. The relative expression of *UGT40A1* gene was low in the 1st to 3rd instars and high in the 4th to 5th instars, indicating that the gene was mainly expressed in the large silkworm period (Figure 4).

**Figure 5:**
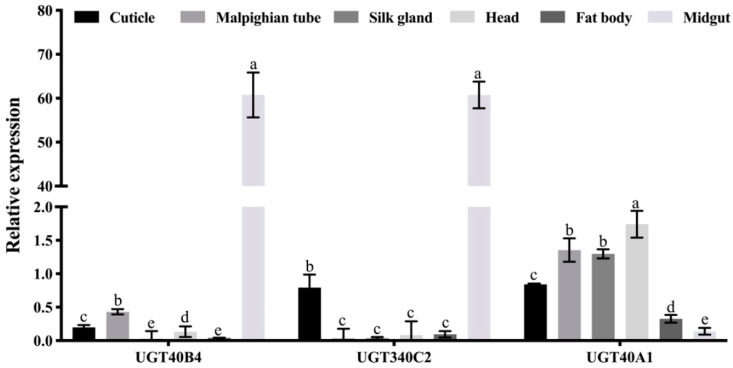
*UGT* gene expression spectrum. Data represent three biological repetitive mean +/− standard deviation, according to Duncan’s multiple range test, to list the different letter’s mean significant difference (*p* < 0.05).

In the tissue expression profile, the relative expression of *UGT40B4* gene was higher in the Malpighian tube and midgut of the silkworm, indicating that the gene may be related to the digestion and metabolism of the silkworm. The relative expression of *UGT340C2* gene in the epidermis and midgut of silkworms was higher, which may be related to the digestion and metabolism of silkworms and the construction of the epidermis. The *UGT40A1* gene is highly expressed in the epidermis, Malpighian tubules, silk glands and head of the silkworm, and may have multiple functions (Figure 5).

### 2.6. Analysis of the Effect of Adding Soybean Isoflavones Test

**Figure 6:**
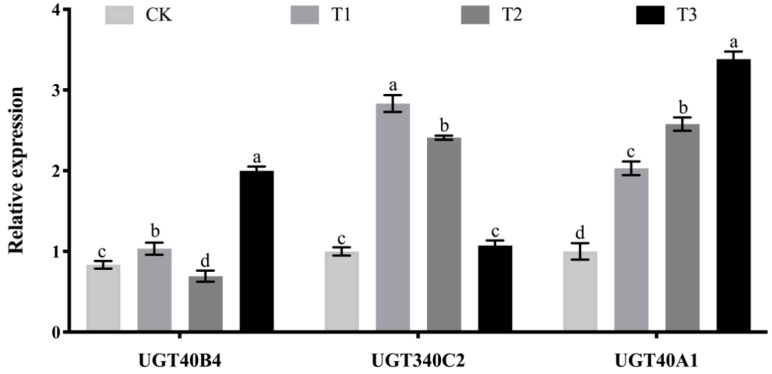
Expression level of *UGT40B4*, *UGT340C2*, *UGT40A1* genes at silkworm fed with soybean isoflavone detected by qPCR. Data represent three biological repetitive mean +/− standard deviation, according to Duncan’s multiple range test, to list the different letter’s mean significant difference (*p* < 0.05).

After adding soybean isoflavones, the expression of *UGT40B4* gene showed a trend of first increasing, then decreasing and then increasing with the increase of soybean isoflavones, indicating that soybean isoflavones could induce the gene expression to a certain extent. The expression of *UGT340C2* gene was significantly higher than that of the control group in the experimental group with 0.1% soybean isoflavones (Figure 6). With the increase of soybean isoflavones in the semi-synthetic feed of the experimental group, the expression of *UGT340C2* gene decreased gradually, indicating that soybean isoflavones in a certain content range could induce the expression of *UGT340C2* gene. With the increase of soybean isoflavone content, the induced expression of *UGT340C2* gene was inhibited. The expression of *UGT40A1* gene increased with the increase of isoflavone content (Figure 6), indicating that soybean isoflavones could significantly induce the expression of this gene.

In the investigation of the body weight of silkworms during the full-eating period, it was found that the addition of 0.1% and 0.4% soybean isoflavones in semi-synthetic feed had a significant negative effect on the body weight of silkworms during the full-eating period (Table 1), indicating that soybean isoflavones had a significant effect on the growth and development of the fifth instar of the silkworm. In the investigation of silkworm cocoon quality, it was found that the cocoon shell weight of the experimental group with soybean isoflavones was improved to a certain extent compared with the control group, and the pupal weight of the experimental group was significantly increased compared with the control group (Table 1), indicating that the addition of soybean isoflavones in the feed can increase the pupal weight.

### 2.7. Analysis of the Effect of Adding Tannic Acid Test

**Figure 7:**
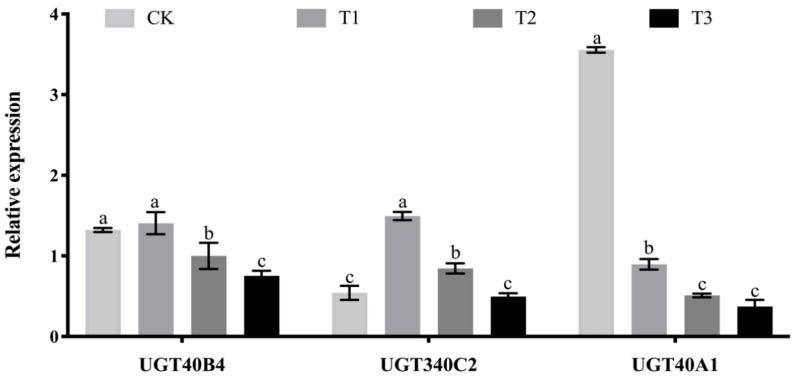
Expression level of *UGT40B4*, *UGT340C2* and *UGT40A1* genes at silkworm fed with tannic acid detected by qPCR. Data represent three biological repetitive mean +/− standard deviation, according to Duncan’s multiple range test, to list the different letter’s mean significant differences (*p* < 0.05).

The test of adding tannic acid to semi-synthetic feed was analyzed. In the test group of adding 0.2% tannic acid to semi-synthetic feed, there was no significant difference in the expression of *UGT40B4* gene compared with the control group (Figure 7). With the increase of tannic acid content in the test group, the expression of this gene showed a downward trend, indicating that tannic acid could inhibit the expression of this gene. The expression of *UGT340C2* gene in the experimental group with 0.2% and 0.8% tannic acid was significantly higher than that in the control group, indicating that low tannic acid content could induce the expression of the gene. The expression of the gene decreased with the increase of tannic acid content in the semi-synthetic feed of the experimental group, indicating that the increase of tannic acid content in the feed could strengthen the inhibition of the gene expression. The expression level of *UGT40A1* gene in the experimental group was significantly lower than that in the control group (Figure 7). With the increase of tannic acid addition in the experimental group, the expression level decreased, indicating that tannic acid could inhibit the expression of this gene.

In the investigation of the body weight of silkworms during the feeding period, it was found that the body weight of silkworms decreased gradually with the increase of tannic acid content in the feed, indicating that tannic acid would have an adverse effect on the growth and development of silkworm in the 5th instar (Table 2). In the investigation of silkworm cocoon quality, it was found that the addition of 0.2% tannic acid to the semi-synthetic feed significantly improved the cocoon quality, and the cocoon quality in the test group with 0.8% tannic acid significantly deteriorated. In the test group with 3.2% tannic acid, silkworms could not normally cocoon or thin cocoons, which seriously affected the quality of cocoons (Table 2), indicating that tannic acid in artificial feed and mulberry leaves had adverse effects on the growth and development of silkworms and the cocooning process of silkworms.

### 2.8. Analysis of the Effect of Adding Arabinoxylan Test

**Figure 8:**
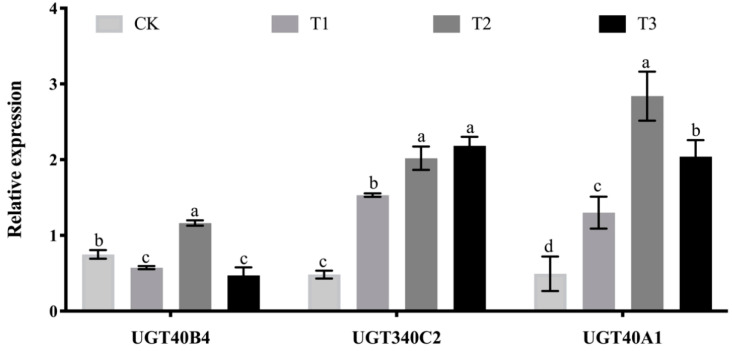
Expression level of *UGT40B4*, *UGT340C2*, *UGT40A1* genes at silkworm fed with arabinoxylan detected by qPCR. Data represent three biological repetitive mean +/− standard deviation, according to Duncan’s multiple range test, to list the different letter’s mean significant differences (*p* < 0.05).

The expression of *UGT40B4* gene decreased first, then increased and then decreased with the increase of arabinoxylan in semi-synthetic feed, indicating that arabinoxylan could induce the expression of this gene to a certain extent (Figure 8). The expression of *UGT340C2* gene increased significantly with the increase of arabinoxylan in semi-synthetic feed, indicating that arabinoxylan could induce the expression of *UGT340C2* gene. There was no significant difference in the expression of *UGT340C2* gene between the experimental groups with 0.4% and 1.6% arabinoxylan. The expression of *UGT40A1* gene increased gradually between 0% and 0.4% of arabinoxylan, indicating that arabinoxylan could induce the expression of the gene. The expression of the gene decreased after adding 1.6% of arabinoxylan (Figure 8), indicating that the high content of arabinoxylan inhibited the expression of the gene to a certain extent.

In the investigation of the body weight of silkworms during the feeding period, it was found that the addition of 0.1% and 0.4% arabinoxylan to the semi-synthetic feed was beneficial to the growth and development of the 5th instar silkworm, and the addition of 1.6% arabinoxylan had a significant adverse effect on the growth and development of the 5th instar silkworm. In the investigation of cocoon quality, it was found that the addition of 0.1% arabinoxylan could increase the cocoon shell weight and pupal weight. After adding 0.4% arabinoxylan, the cocoon quality was not significantly different from that of the control group (Table 3). After adding 1.6% arabinoxylan, the cocoon quality has significantly deteriorated, indicating that an appropriate amount of arabinoxylan could improve the production performance of silkworms to a certain extent (Table 3). The high content of arabinoxylan would have a toxic effect on silkworms and affect the growth and development of silkworms.

## 3. Discussion

Uridine diphosphate-glycosyltransferases (UGTs) are pivotal multifunctional detoxification enzymes that participate in the metabolism of xenobiotic toxic substances [14,22,23]. These enzymes play a crucial role in insects’ metabolism and elimination of plant-derived toxic compounds and exogenous noxious substances [24,25,26]. Concurrently, insect UGTs also fulfill important functions in various physiological processes [27,28,29,30]. Previous studies have shown that UDP-glycosyltransferase (UGT) catalyzes the binding of a series of different lipophilic small compounds to sugars to produce glycosides, and plays an important role in the detoxification of xenobiotics and the regulation of insect endogens [18]. Other scholars revealed the UDP - glycosyl transferase (UGT) and phospholipase gene in flavonoids and the important role of glycerol phospholipid metabolism [31].

Upon supplementing semi-synthetic feed with soybean isoflavones, tannins, and arabinoxylan, we observed differential expression of *UGT40B4*, *UGT340C2*, and *UGT40A1*(Table 1, Table 2 and Table 3). Notably, soybean isoflavones induced the expression of *UGT340C2* and *UGT40A1* genes. Furthermore, tissue expression profiles revealed elevated expression of *UGT40B4* and *UGT40A1* genes in the midgut of silkworms reared on artificial diet. Study found that in the diet adding 20 mg/kg and 80 mg/kg of soybean isoflavones not only can improve the growth performance, but also be beneficial to the immune response to poultry [32], the addition of soybean isoflavones to semi-synthetic feed exerted varied effects on silkworm growth, development, cocoon quality, and pupal mass, suggesting differential requirements for soybean isoflavones at various stages of silkworm development. By analyzing the transcriptome and proteomics of salivary gland function genes and oral secretion (OS) protein changes of *Helicoverpa armigera* fed with artificial diet (containing gossypol and tannin) and cotton plant leaves, it was found that cotton leaves, gossypol and tannin can significantly up-regulate the *GST*, *UGT*, hydrolase and lipase genes of *Helicoverpa armigera*, which are involved in the detoxification and digestion of *Helicoverpa armigera* [33]. The tannic acid content in mulberry leaves was about 1.8~2.9%, the tannic acid content in M38 artificial diet was about 0.6~1.2%. The tannic acid content in mulberry leaves was significantly higher than that in artificial diet, and tannic acid could induce the expression of *UGT340C2* gene. It is speculated that *UGT340C2* gene may be involved in the detoxification function of the silkworm in artificial diet, and may also be related to the tannic acid metabolism process.

Research findings indicate that the UGT013829 gene in silkworms enables flavonoids to undergo glycosylation. Moreover, this gene is involved in both olfactory responses and detoxification processes [17]. Among the 52 *UGT* genes of *Spodoptera litura*, the enzyme activity and transcription level of 77% UGT members were significantly up-regulated after flavonoid treatment. The bacteria co-expressing UGTs had a higher survival rate under flavonoid treatment, and flavonoids were significantly metabolized by UGT recombinant cells, indicating that UGTs were involved in flavonoid detoxification [34]. Through molecular docking, we discovered potential interactions between the three UGT proteins in our experiment and soybean isoflavones. Consequently, we hypothesize that these three UGT proteins may glycosylate soybean isoflavones, thus participating in their metabolism and absorption processes [35,36].

Through proteomic analysis of the midgut in artificially reared and mulberry leaf-fed silkworms, we identified 964 significantly differentially expressed proteins. *UGT40B4*, *UGT340C2*, and *UGT40A1* exhibited distinct expression patterns across developmental stages and tissues. Molecular docking techniques revealed that *UGT40B4*, *UGT340C2*, and *UGT40A1* proteins demonstrated strong binding affinities for isoflavones, tannins, and arabinoxylans. The expression of three *UGT* genes in silkworms was significantly up-regulated by adding soybean isoflavones to semi-synthetic feed. Tannins induced the expression of the *UGT340C2* gene, while *UGT340C2* and *UGT40A1* were significantly upregulated in the arabinoxylan-supplemented group. Tannin present in artificial diets and mulberry leaves adversely affected silkworm growth, development, and cocoon formation. Moderate amounts of arabinoxylans positively influenced the growth, development, and cocoon quality of fifth-instar silkworms. However, high concentrations of arabinoxylans exhibited toxic effects, impacting silkworm growth and development.

## 4. Materials and Methods

### 4.1. Test Silkworm Varieties and Feed

The test silkworm variety is the No.1 silkworm strain of Youshi No.1 bred by the laboratory. The mulberry leaves were from the Forestry Experimental Base of Panhe Campus of Shandong Agricultural University, and the variety was Nongsang 14. The feed used is the M38 cooking artificial feed developed and processed by the laboratory. The main components of M38 artificial feed used in this experiment were mulberry leaf powder 38%, soybean meal 30%, corn flour 24.3%, citric acid 3.0%, inorganic salt 2%, compound VB 0.3%, Vc 2%, preservative 0.4%. An appropriate amount of artificial feed powder was weighed, and 1.8 times the weight of the powder was added with purified water. After fully mixed, it was cooked at 100 °C for 60 min. After cooling to room temperature, it was placed in a refrigerator at 4 °C for later use.

### 4.2. Sample Processing Materials

The midgut of the 5th instar female silkworm reared on an artificial diet and mulberry leaves at 72 h were taken, and the peritrophic membrane was removed. The food residue was rinsed in 1 × PBS and placed on ice. The washed midgut epithelium was cut longitudinally along the midline and quickly placed in a 1.5 mL RNase-free centrifuge tube. Three half midguts from different individuals were placed in each tube, and three replicates were set for each group. The centrifuge tube was quickly frozen in liquid nitrogen and stored in a refrigerator at −80 °C for later use.

### 4.3. Fluorescence Quantitative PCR

RNA was extracted from samples using TransZol Up RNA extraction reagent (Beijing all-gold Company, Beijing, China). The first strand of cDNA was synthesized using the EasyScript ^®^ One-Step gDNA Removal and cDNA Synthesis SuperMix (Beijing All-Style Gold Company, Beijing, China) kit. The qPCR reaction system and conditionsThe qPCR instrument was Bio-Rad CFX96, and TransStart ^®^ TipGreen qPCR SuperMix (Beijing TransGen, Beijing, China) kit was used. The reaction conditions were as follows: pre-denaturation at 94 °C for 30 s, denaturation at 94 °C for 5 s, annealing at 55 °C for 15 s, extension at 72 °C for 10 s, 40 cycles.

### 4.4. Semi-Synthetic Artificial Feed Feeding Test Materials

The basic semi-synthetic feed formula in the feeding test was designed. The main components of the semi-synthetic feed are soybean protein (30%), cellulose powder (24.0%), corn starch (16%), sucrose (10%) and agar (10%), and contain small amounts of vitamins and inorganic salts. The tested silkworm varieties were the same as 2.1. Females and males were identified when the 5th instar larvae were raised. Females at 72 h of the 5th instar were used in the experiment. The 1st to 4th instar silkworms were fed with M38 artificial diet. After the 5th instar silkworms were raised, semi-synthetic diets with different contents of soybean isoflavones, tannic acid and arabinoxylan were used for feeding. Three replicate groups were set up under three content gradients, with 50 female silkworms in each group. The appropriate amount of powder semi-synthetic artificial feed was weighed, and pure water with 1.8 times the weight of the powder was added. After mixing, it was put into a point-broken bag, cooked at 100 °C for 60 min, pressed flat before cooling, and cooled to room temperature. Feed or store in a refrigerator at 4 °C.

### 4.5. Experiment on Adding Soybean Isoflavones to Semi-Synthetic Feed

In the isoflavone supplementation experiment, four experimental groups were set up. The semi-synthetic diet in the control group was not supplemented with soy isoflavones. The semisynthetic diet of group 1 was supplemented with 0.1% soy isoflavones; The semi-synthetic diet of the two groups was supplemented with 0.4% isoflavones; The semi-synthetic diets of three groups were supplemented with 1.6% soy isoflavones. Sheng food to 5 age period of mulberry silkworm body quality measurement, the age of 5. 72 h of the experimental group in the silkworm intestine samples for fluorescence quantitative analysis of the amount of *UGT* gene expression.

### 4.6. Test of Adding Tannic Acid in Semi-Synthetic Feed

In the experiment of adding tannic acid, the control group did not add tannic acid in the semi-synthetic feed of the feeding control group; the semi-synthetic feed of the experimental group 1 was added with 0.2% tannic acid; the semi-synthetic feed of the experimental group 2 was added with 0.8% tannic acid; in the experimental group 3, 3.2% tannic acid was added to the semi-synthetic feed. The body weight of silkworms in the 5th instar feeding period was measured, and the expression of *UGT* genes in the midgut samples of silkworms in each experimental group at 72 h of the 5th instar was quantitatively analyzed by fluorescence.

### 4.7. Experiment on Adding Arabinoxylan to Semi-Synthetic Feed

In the experiment of adding arabinoxylan, the control group did not add arabinoxylan in the semi-synthetic feed of the feeding control group; the semi-synthetic feed of the experimental group 1 was supplemented with 0.1% arabinoxylan; the semi-synthetic feed of the experimental group 2 was added with 0.4% arabinoxylan; 1.6% arabinoxylan was added to the semi-synthetic feed of the experimental group 3. The body weight of silkworms in the 5th instar feeding period was measured, and the expression of *UGT* genes in the midgut samples of silkworms in each experimental group at 72 h of the 5th instar was quantitatively analyzed by fluorescence.

### 4.8. Statistical Analysis

Biological replicates were conducted a minimum of three times, and the results are presented as the mean ± SD. *p*-values were calculated using Student’s *t*-test for two samples and One-way ANOVA (Tukey’s HSD test) for comparisons involving more than two samples, utilizing Prism 8 (GraphPad, San Diego, CA, USA) and SPSS Statistics 26.0 software (SPSS Inc., Chicago, IL, USA).

## Figures and Tables

**Table 1:** Effects of soybean isoflavones supplementation on body weight of silkworm 5th instar and cocoon quality.

Group	Weight (g)	Cocoon Weight (g)	Cocoon Layer Weight (g)	Pupae Weight (g)	Cocoon Layer Rate (%)
CK	15.21 a	1.41 d	0.28 c	1.13 d	19.61 a
T1	11.35 b	1.48 c	0.29 bc	1.19 c	19.73 a
T2	11.98 b	1.57 b	0.31 b	1.26 b	19.80 a
T3	15.97 a	1.64 a	0.32 a	1.32 a	19.62 a

Note: CK means control check, T1 means treatment 1, T2 means treatment 2, T3 means treatment 3. According to Duncan’s multiple range test, to list the different letter’s mean significant difference (*p* < 0.05).

**Table 2:** Effects of tannic acid supplementation on body weight of silkworm 5th instar and cocoon quality.

Group	Weight (g)	Cocoon Weight (g)	Cocoon Layer Weight (g)	Pupae Weight (g)	Cocoon Layer Rate (%)
CK	19.09 a	1.45 b	0.25 b	1.20 b	17.76 a
T1	18.54 b	1.60 a	0.30 a	1.30 a	18.70 a
T2	14.51 c	1.36 c	0.21 c	1.15 b	15.50 a
T3	12.72 d				

Note: CK means control check, T1 means treatment 1, T2 means treatment 2, T3 means treatment 3. The treatment 3 were all thin-layer cocoons or no cocoons, and there was no available data. According to Duncan’s multiple range test, to list the different letter’s mean significant difference (*p* < 0.05).

**Table 3:** Effects of arabinoxylan supplementation on body weight of silkworm 5th instar and cocoon quality.

Group	Weight (g)	Cocoon Weight (g)	Cocoon Layer Weight (g)	Pupae Weight (g)	Cocoon Layer Rate (%)
CK	15.21 c	1.41 b	0.28 b	1.13 b	19.61 a
T1	16.41 b	1.53 a	0.31 a	1.22 a	19.85 a
T2	17.99 a	1.41 b	0.27 b	1.14 b	19.39 a
T3	11.35 d	1.29 c	0.25 c	1.04 c	19.63 a

Note: CK means control check, T1 means treatment 1, T2 means treatment 2, T3 means treatment 3. According to Duncan’s multiple range test, to list the different letter’s mean significant difference (*p* < 0.05).

## Data Availability

All data support the results of this study can be in online and its Supplementary Data obtained in the paper.

## Supplementary Materials

The following supporting information can be downloaded at: https://www.mdpi.com/article/10.3390/ijms26031309/s1.
