# Supplementary material for: Proteomic Analysis of Midgut of Silkworm Reared on Artificial Diet and Mulberry Leaves and Functional Study of Three UGT Genes
Source: Int J Mol Sci. 2025 Feb 4;26(3):1309. doi: 10.3390/ijms26031309 (PMC11818671; doi:10.3390/ijms26031309)
Supplement: Supplementary file 1 [file ijms-26-01309-s001.zip › ijms-3410773-supplementary.pdf]

Table S1. Primers for real-time PCR.

Gene symbol	primer
UGT40B4-F	GCACCACAGCAAAGCATA
UGT40B4-R	TTTCCGTTGTAGACAATAAGC
UGT340C2-F	TTGGAATCGGTATCAGTTTG
UGT340C2-R	GATCGTGCATCAGGGTTTT
UGT40A1-F	CGGCTTACTGTCTACAACTGA
UGT40A1-R	CAACTTCGAGGGCAAATC
RP49-F	TCAATCGGATCGCTATGACA
RP49-R	ATGACGGGTCTTCTTGTTGG

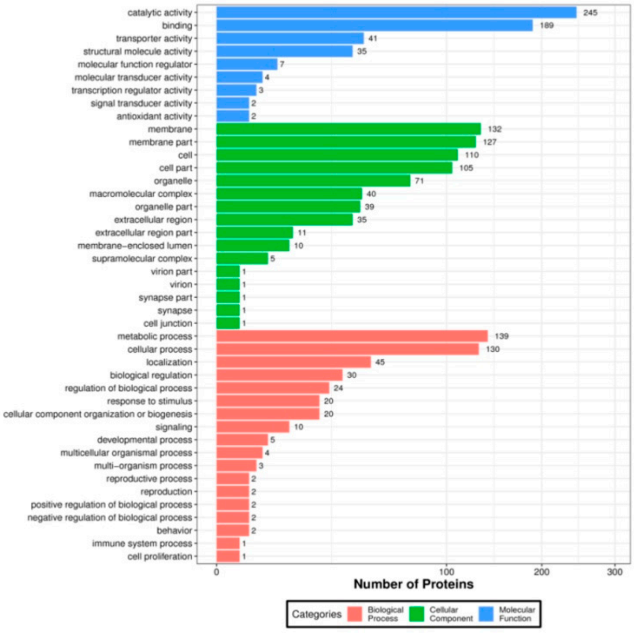

Figure S1. GO Functional classification of differentially expressed protein.

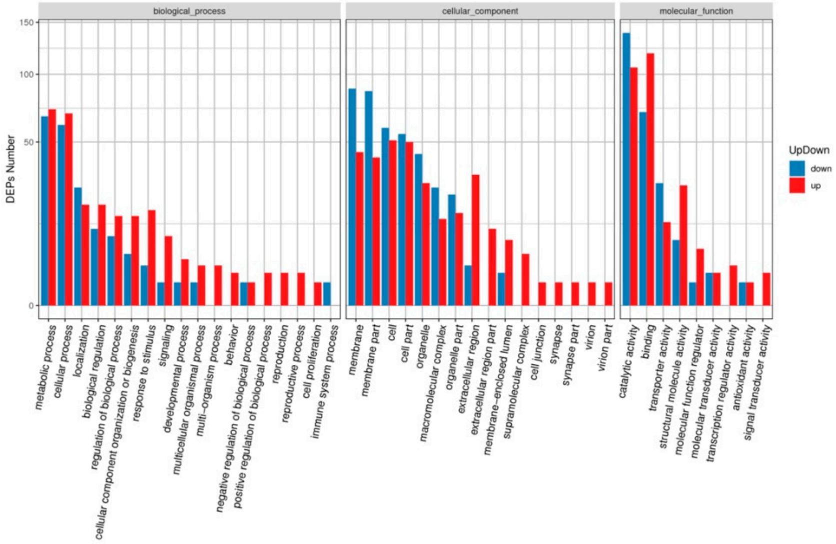

Figure S2. GO enrichment bubble chart of differentially expressed proteins.

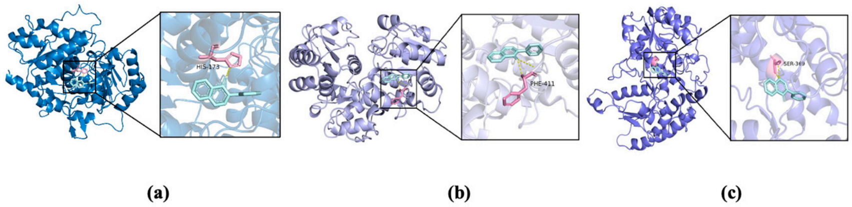

Figure S3. Schematic diagram of the molecule docking structure of UGT40B4, UGT340C2, UGT40A1 and soybean isoflavone. (a) Schematic diagram of the molecular docking structure of UGT40B4 with soy isoflavone (b) Schematic diagram of the molecular docking structure of UGT340C2 with soy isoflavone (c) Schematic diagram of the molecular docking structure of UGT40A1 with soy isoflavone.

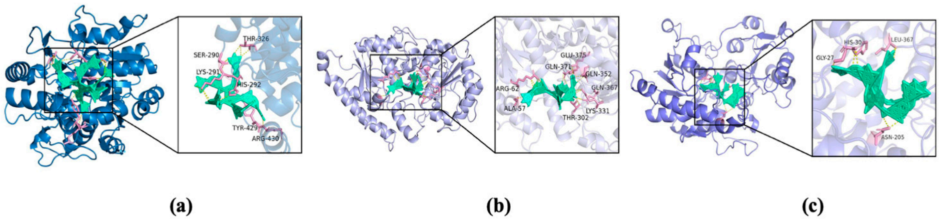

Figure S4. Schematic diagram of the molecule docking structure of UGT40B4, UGT340C2, UGT40A1 and tannic acid. (a) Schematic diagram of the docking structure of UGT40B4 with tannic acid molecules (b) Schematic diagram of the docking structure of UGT340C2 with tannic acid molecules (c) Schematic diagram of the docking structure of UGT40A1 with tannic acid molecules.

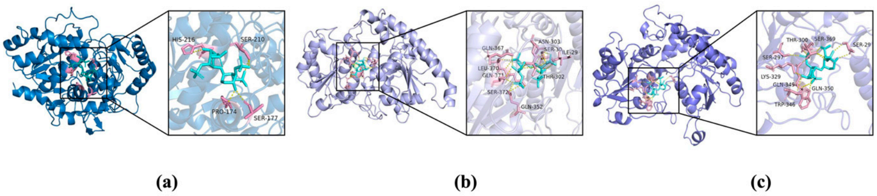

Figure S5. Schematic diagram of the molecule docking structure of UGT40B4, UGT340C2, UGT40A1 and arabinoxylan. (a) With the arabinoxylan UGT40B4 molecular docking structure diagram (b) With the arabinoxylan UGT340C2 molecular docking structure diagram (c) With the arabinoxylan UGT40B4 molecular docking structure diagram.
